# Dendritic Cells in the Gastrointestinal System: Division of Labor, Plasticity, and Niche‐Specific Adaptation

**DOI:** 10.1111/imr.70090

**Published:** 2025-12-25

**Authors:** Yixuan D. Zhou, Macy R. Komnick, Daria Esterházy

**Affiliations:** ^1^ Department of Pathology University of Chicago Chicago Illinois USA; ^2^ Committee on Immunology University of Chicago Chicago Illinois USA

**Keywords:** dendritic cells, digestive system, gut draining lymph nodes, tissue specific immunity

## Abstract

The intestinal immune system is constantly challenged to distinguish between innocuous dietary antigens, commensal microbiota and intestinal self‐antigens (self‐Ags) versus harmful pathogens and malignant cells. It resides in the intestinal wall itself, Peyer's patches (PPs) and the draining lymph nodes (LNs). Dendritic cells (DCs) are found in all of these structures and are professional antigen‐presenting cells (APCs) dictating and maintaining T cell fate. Here, we review how DCs contribute to immune homeostasis in the gastrointestinal system through multiple strategies: division in labor and strategic anatomical positioning between DC subtypes, plasticity and site‐specific functional adaptation. While these properties of DCs are likely not unique to the gastrointestinal tract, it is the site where we have learned most about how this DC network operates.

**Abbreviations**
APC: antigen‐presenting cell
cDC: conventional DC
DC: dendritic cell
gLN: gut‐draining LN
LN: lymph node
LP: lamina propria
pDC: plasmacytoid DC
PP: Peyer's patch
pTreg cell: peripherally induced regulatory T cell
RA: retinoic acid

## 1 Introduction

The intestinal immune system is constantly challenged to distinguish between innocuous dietary antigens, commensal microbiota, and intestinal self‐antigens (self‐Ags) versus harmful pathogens and malignant cells. It resides in the intestinal wall itself, Peyer's patches (PPs), and the draining lymph nodes (LNs). Dendritic cells (DCs) are found in all of these structures and are professional antigen‐presenting cells (APCs) dictating and maintaining T cell fate. They thus occupy a central role in orchestrating the delicate balance between tolerance, defense, and tissue repair in the gut, failure of which leads to diseases such as allergies, chronic inflammation, and cancer. In this review, we discuss how DCs contribute to immune homeostasis in the digestive system, on the one hand through division of labor between ontologically and functionally distinct DC subsets, and on the other by DC remarkable plasticity and adaptation to a given tissue environment.

## 2 Divide et Immuna: Complementary Specialization of Intestinal DC Subsets

Recent advances in single‐cell technologies and genetic lineage tracing have revealed extensive heterogeneity among intestinal DC subsets [1, 2, 3, 4]. This heterogeneity reflects a sophisticated division of labor in the DC compartment based on cellular ontogeny, tissue localization, phenotypic specialization, and functional capacity. In this section, we synthesize the current understanding of intestinal DC diversity with a focus on how this division of labor enables fine‐tuned adaptive responses.

### 2.1 Cell Type Division: Ontogeny of Intestinal DCs

**FIGURE 1:**
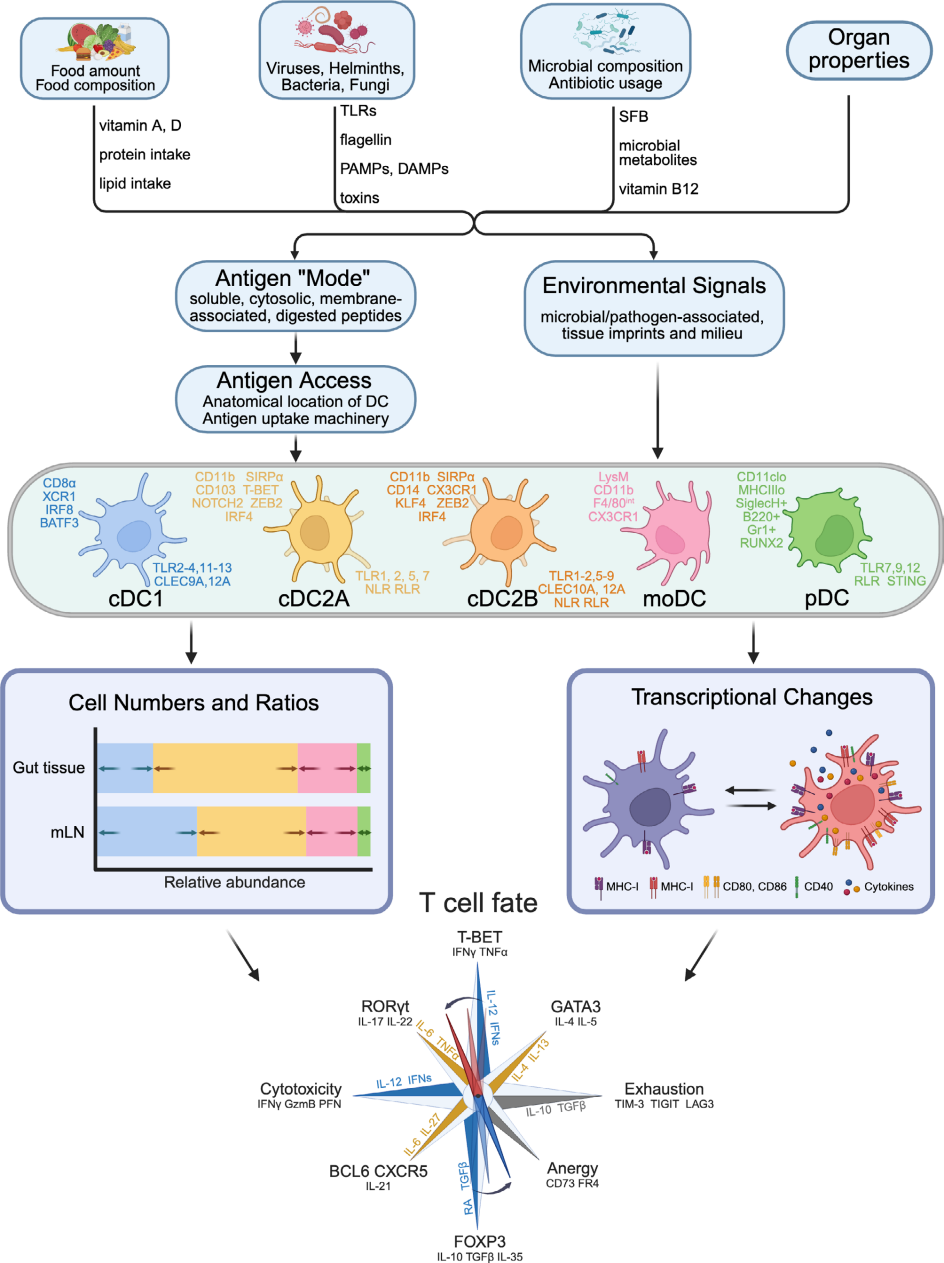
The DC pool integrates complex environmental cues to shape its composition and phenotype and directs T cell differentiation. Schematic overview of how environmental signals influence intestinal DC populations and subsequent T cell fates. Various environmental factors including diet (food amount and composition, vitamins A and D, protein and lipid intake), microbiota (microbial composition, antibiotic usage), intestinal pathogens (viruses, helminths, bacteria, fungi), and tissue imprints provide diverse and complex signals to intestinal DCs. The integration of these signals influences the total numbers and the composition of major DC subsets (cDC1, cDC2A, cDC2B, moDC and pDC), as well as the transcriptional profiles, especially activation markers (MHC‐I, MHC‐II, CD80, CD86, CD40) and cytokine production, of those cells in gut tissue and gLN. These DC‐mediated changes ultimately determine T cell differentiation pathways, promoting distinct T cell fates including T‐BET+ Th1 cells (IFNγ, TNFα), GATA3+ Th2 cells (IL‐4, IL‐5), RORγt+ Th17 cells (IL‐17, IL‐22), BCL6+ Tfh cells (CXCR5, IL‐21), FOXP3+ Treg cells (IL‐10, TGFβ, IL‐35), cytotoxic T cells (IFNγ, GzmB, PFN), exhausted T cells (TIM‐3, TIGIT, LAG3), and anergic T cells (CD73, FR4).

Intestinal DCs arise from distinct developmental pathways that give rise to functionally specialized subsets (Figure 1). Conventional DCs (cDCs) are the most prominent ones, which are divided into cDC1 and cDC2 lineages. Both are derived from a common DC precursor (CD115^+^ CDPs) in the bone marrow.

cDC1s can typically be identified by being XCR1^+^SIRPα^−^CD11b^−^, and a subpopulation is marked by additionally expressing CD8α. Markers like CD24 and DEC205 are also used to identify this cell type. The known transcription factors that govern the development of cDC1s from CDPs are IRF8, BATF3, and ID2 [5]. cDC1s are equipped with potent antigen processing and presentation machinery for extracellular antigens and therefore excel at cross‐presentation via MHC‐I molecule [6], an essential step for the initiation of CD8^+^ T cell responses against viral infection and malignancies. The necessity of cDC1s for CD8^+^ T cell priming was confirmed by using *Batf3* ^−/−^ or *Irf8* ^CD11cΔ^ mice [7]. However, recent studies have discovered that *Batf3* ^−/−^ mice contain a residual population of CD8α^+^ DC [8] as well as a population of cDC1‐like DCs that co‐express cDC2 markers [9], and the most complete and selective ablation of the cDC1 lineage to date is achieved in a mouse lacking an IRF8 enhancer element (*Irf8*+*41* ^−/−^ mouse) [10]. cDC1s are also known for their ability to balance between T helper 1 (Th1) and T regulatory (Treg) cell induction in the periphery upon environmental stimuli through the regulation of transforming growth factor beta (TGF‐β) production and retinaldehyde dehydrogenase (RALDH) activity [1, 3].

In contrast, cDC2s, identifiable by flow cytometry as CD11b^+^XCR1^−^SIRPα^+^, preferentially drive Th2 and Th17 responses and are the primary DCs supporting T follicular helper cell (Tfh) development [11]. cDC2 development depends on the transcription factor IRF4 [12], though more efficient cDC2 ablation has recently been achieved through deletion of three NFIL3 binding sites in the promoter of another transcription factor, ZEB2 (*Zeb2* ^Δ1+2+3^ mice) [13]. Compared to cDC1s, cDC2s exhibit greater versatility in cytokine production and are particularly effective at initiating immune responses against extracellular pathogens, including bacteria, helminth, and fungi. While cDC2s can also induce Treg cells, their efficiency in doing so is generally lower than that of cDC1s [3]. The versatility in cDC2 function has recently also been attributed to the existence of cDC2s sublineages; one depends on transcription factor KLF4, the other on transcriptional co‐activator NOTCH2. NOTCH2‐dependent cDC2s are essential for inducing Th17 cells to support mucosal immunity and tissue repair, while KLF4‐dependent cDC2s promote Th2 differentiation, especially during helminth infection or allergic inflammation [14, 15]. Complementarily, recent single‐cell resolution work has resolved cDC2 heterogeneity using a functional and transcriptomic axis based on the expression of T‐bet and RORγt. This newer framework classifies cDC2s into two distinct subsets: cDC2A (T‐bet^+^, anti‐inflammatory) and cDC2B (RORγt^+^, TNF‐ and IL‐6‐producing, pro‐inflammatory) [16]. A follow up study demonstrated that the fate specification of cDC2A and cDC2B starts in the bone marrow by having distinct precursors, with cDC2As being NOTCH2 and cDC2Bs KLF4 dependent [9], suggesting that cDC2 subsets are ontogenetically determined lineages with different functional specialization. This ontogenic framework is useful, as cDC2s display very distinct expression patterns depending on the tissue in which they sit, making their analysis by a universal cell surface marker panel challenging. Recent scRNAseq of gut DCs revealed that some of the best surface markers to distinguish cDC2A from cDC2B there may be NOTCH2^+^CD103^hi^CLEC4A^+^ (cDC2A) and CCR2^+^SIRPα^hi^CX3CR1^+^TMEM176A/B^+^ (cDC2B) [17]. Similarly, the classification into being anti‐versus pro‐inflammatory described in the spleen may not be appropriate in the context of the gut, where cDC2As support type 3 immunity [18, 19] while cDC2Bs type 2 immunity [15], and a group has even identified a third, infection induced (inf‐cDC2) subset in the lung of which it is not fully clear yet if it has its own precursor or branches off from the cDC2A, cDC2B or both lineages [20].

Plasmacytoid DCs (pDCs), derived from a separate precursor called common lymphoid progenitor, are marked by low CD11c and MHCII expression and are Siglec‐H^+^B220^+^Gr‐1/Ly6C^+^ [21]. Unlike cDCs whose precursors leave the bone marrow and differentiate in the periphery, pDCs fully differentiate in the bone marrow and migrate in an E2‐2 and RUNX2‐dependent manner [22]. They are relatively rare in frequency in the gut and are notable for their robust production of type I interferons during viral infections, while they are less effective at canonical antigen presentation [2]. Their role in intestinal immunity is less well understood, although they are believed to contribute to pathogen defense and influence the local immune environment through cytokine production.

Another major APC type in the gut is monocyte‐derived DC (moDC, also referred to as DC3) [23], which infiltrates the gut during inflammation, contributing to both pathogen clearance and immune regulation. Of note, monocyte‐derived *macrophages* are also abundant in the gut, exhibiting similar antigen‐presentation ability and surface markers as moDCs, though their in‐depth discussion is out of the scope of this review. moDCs in the gut can be identified as CD11b^+^CX3CR1^+^CD64^int^F4/80^int^ [24]. They are particularly relevant during episodes of intestinal inflammation, such as in inflammatory bowel disease (IBD) and Crohn's Disease, where they not only increase in frequency but also adopt a more pro‐inflammatory phenotype [25, 26].

Recent studies have identified a unique lineage of RORγt^+^ APCs (also referred to as Thetis and Janus cells), enriched in the neonatal intestinal immune system, as critical players in pTreg cell induction and the establishment of immune tolerance to food and commensal bacteria derived antigens [27, 28, 29, 30, 31, 32]. These cells appear developmentally distinct from cDCs and may represent an evolutionarily conserved mechanism for promoting immune tolerance to dietary and commensal antigens during the early postnatal window [28, 30], though they remain critical for pTreg cell development in response to these classes of antigens even in adult mice [33]. Unlike cDC1s, whose development depends on BATF3 and IRF8, RORγt^+^ APCs are not affected by *Batf3* deficiency, indicating a separate ontogeny, although their precursor remains to be identified. They are instead dependent on RORγt [31, 34] and, among APCs, uniquely express the transcription factor PRDM16 [34]. RORγt^+^ APCs share features with ILC3s, which are also RORγt‐dependent and in fact were originally classified as a subset of ILC3s that has robust antigen‐presenting and tolerance induction capacity [35]. Similarly, because RORγt^+^ APCs also express CD11c and the transcription factors ZBTB46 and IRF8 [30], DC ablation strategies based on these factors unknowingly also targeted them [3]. RORγt^+^ APCs are predominantly found in lymphoid tissues, including the gut‐draining LNs (gLNs), where they are resident and express a suite of MHC‐II molecules and co‐stimulatory ligands [27, 30, 32, 36].

The proportional representation of each DC subset varies depending on the tissue (Figure 1). In the lamina propria (LP), cDC1s represent 10%–30% and cDC2s 50%–60% of the DC population, though macrophages are overall the dominant APC in the gut (60%–70% of all CD11c^+^ cells) [3]. CD103^+^CD11b^−^ cDC1s represent approximately 15% of the DC population in the PP [37, 38]. However, in the gLNs, cDC1s and cDC2s each make up ~40% of the CD11c^+^ compartment, with the remaining portion filled by moDCs [3] and RORγt^+^ APCs being very rare. Of note, these proportions are based on flow cytometry, and it is possible that due to differential extraction efficiencies some subsets are under‐ or overrepresented. Comprehensive imaging‐based analyses would be required to resolve this issue. The ratios are also not static; they can shift dynamically in response to age, microbial colonization, dietary changes, and local inflammatory cues. For example, in the more distal gLNs, cDC1s can make up to 70% of the migratory DCs while they only represent 40% in the small intestinal gLNs [39]. The development of certain DC types is impaired or delayed in germ‐free mice [40], and helminth infections lead to an exclusion of cDC1s while expanding the two cDC2 subsets in the gLNs [39] and gut [33].

Importantly, DCs traffic into the gut as pre‐DCs and complete their differentiation locally under the influence of tissue‐derived factors such as retinoic acid (RA), TGF‐β, and microbial metabolites [41]. This in situ differentiation and maturation fine‐tunes the functional capacity of DCs to their microenvironment, allowing for rapid adaptation to the immunological demands of the gut. Tissue imprints on DCs are discussed in more depth in the second half of this review.

### 2.2 Phenotypic and Functional Division

As described above, different intestinal DC subsets exhibit distinct surface marker profiles that facilitate their identification by flow cytometry [11]. Many of these commonly used markers already reflect different functions or behavior: for example, XCR1, the chemokine receptor for XCL1 and XCL2, is primarily expressed on cDC1s; the CD47 receptor SIRPα is primarily on cDC2s, regardless of the spatial locations [11]; CX3CR1, the receptor for CX3CL1, guides APC dendrites into the gut lumen [42] and is expressed in a fraction of all gut APC subsets [3]; CCR7 is the receptor for CCL19/21, aiding DC migration from the gut to LNs and is used as a marker for all migratory DCs [43], as is CD103, a subunit of integrin αEβ7, which binds E‐Cadherin on epithelial cells.

**FIGURE 2:**
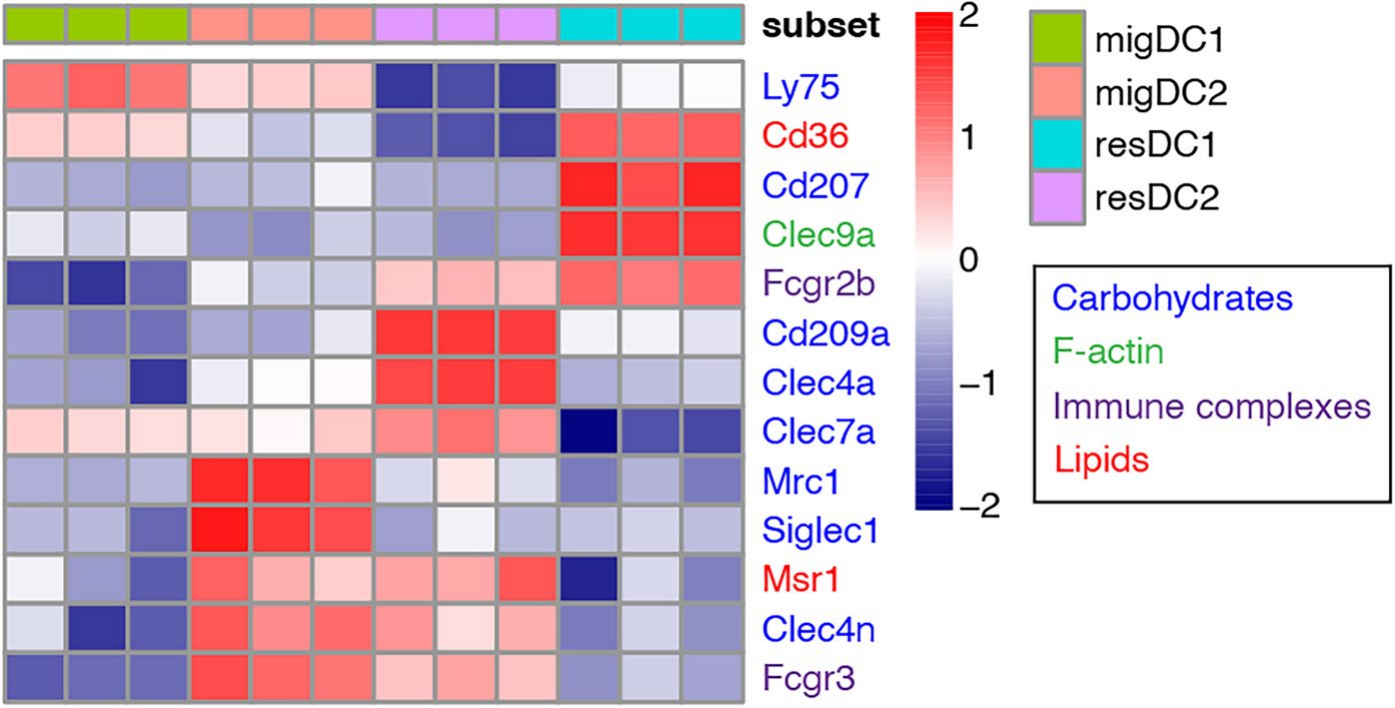
Expression of cargo uptake machinery in gut DCs. Heatmap displaying counts per million (CPM) expression of selected antigen/cargo uptake machinery in different DC subsets sorted from the gLNs (raw data from Esterházy et al., Nature Immunology, 2016). Text color indicates ligand specificity.

These markers were identified long before systematic expression profiling of the DCs in the gut and draining LNs, which has by now been performed [3, 44]. Transcriptome analyses have revealed that the ontogenically distinct intestinal DC subsets also differ in their expression of cargo uptake machineries, microbial sensing and downstream signaling molecules, all the way to their cytokine output, as well as metabolism, resulting in the secretion of immunomodulatory molecules (Figure 1). As such, cDC1s express the pattern recognition receptors (PRRs) TLR2–4, TLR11–13 and C‐type lectin‐like receptor (CLEC)9A and 12A that facilitate the recognition of necrotic cells and enhance cross‐presentation of cell‐associated antigens [3, 24, 45]. In contrast, cDC2s are equipped with a wider range of TLRs [1, 2, 4, 5, 6, 7, 8, 9, 13] and CLECs (4A, 6A, 7A) and also express Nod‐like receptor (NLR) and RIG‐I‐like receptor (RLR), highlighting the more versatile role that they play in intestinal adaptive immunity. Among these, the expression of TLR5 and TLR9 is notably high on cDC2s, making them particularly responsive to flagellated bacteria and unmethylated CpG DNA, respectively [3, 14, 46]. pDCs express TLR7, 9 and 12, as well as RLR and STING, indicating their specialization in controlling viral infections. PRR expression by moDCs is still poorly defined and remains one intriguing area for future studies. In terms of antigen loading, while all DCs have some capacity to pino‐, macro‐, and phagocytose, subsets do express distinct patterns of selective cargo receptors, which in some cases also have PPR function (Figure 2).

Functionally, intestinal DCs orchestrate immune responses through the production of distinct cytokine profiles (Figure 1). cDC1s secrete IL‐12 and type III interferons, supporting Th1 polarization and antiviral immunity. They also contribute to the development of cytotoxic CD8^+^ T cells and are essential for protective responses against intracellular pathogens [45]. cDC2s, by contrast, produce TGF‐β‐inducing MMP9 (cDC2A) or IL‐6 and TNFα (cDC2B), promoting Th2, Th17, or Tfh cell responses that are effective against helminth, extracellular bacteria, and fungi [1, 4]. In addition, both cDC1s and cDC2s can produce TGF‐β and RA, particularly in the steady‐state, to drive the differentiation of FOXP3^+^ Treg cells and promote oral tolerance. However, cDC1s are superior to cDC2s in this function, which is explained by their higher expression, compared to cDC2s, of *Aldh1a2*, encoding RALDH2, the rate limiting enzyme for generating RA from Vitamin A, and genes necessary for efficient TGF‐β exposure [3]. While the precise role of cDC1s in tolerance to dietary and microbial antigens has been debated since the recent discovery of RORγt^+^ APCs [27, 28, 29, 30, 31], cDC1s are known to play a role in breaking oral tolerance upon intestinal viral infection [47, 48] through increased production of IL‐12 and subsequent Th1 differentiation of oral antigen specific CD4^+^ T cells. Additionally, cDC1s have been shown by us and others to be critical for tolerance to antigens derived from intestinal epithelial cells [49, 50]. In contrast, while it is clear that RORγt^+^ APCs seem absolutely required for pTreg cell induction in response to dietary and bacterial antigens, we recently found that they are dispensable for pTreg cell induction in response to gut self‐antigens [50]. Regardless, the discovery of RORγt^+^ APCs expands the landscape of tolerogenic APCs in the gut immune system and suggests that tolerance may be orchestrated by multiple APC lineages.

pDCs primarily produce Type 1 interferons and display less crosstalk with other immune cell types. However, some recent studies have described their extensive interaction with innate lymphocytes (ILCs) and T cells [21]. pDCs have also been implicated in the induction of oral tolerance [51], though the study did not show direct induction of Treg cells by pDCs.

### 2.3 Spatial Division: Tissue Versus Lymph Node DCs

A significant dimension of intestinal DC heterogeneity arises from their spatial localization, particularly the functional dichotomy between those residing in the LP and those located in the gLNs. DCs in the LP and PPs are strategically positioned to monitor luminal contents through forming transepithelial dendrites to sample luminal oral or microbial antigens [42, 52, 53] and interacting with specialized epithelial cells [54]. LP DCs are primarily responsible for capturing dietary and microbial antigens and initiating local tolerance or immune activation in a context‐dependent manner. CD103, also known as Integrin alpha E, is important for cell adhesion and migration. CD103^+^ DCs, which could be CD11b^−^ (cDC1s) and CD11b^+^ (cDC2s), are generally thought to be migratory DCs (migDCs) that migrate from the gut to gLNs via afferent lymphatics. These migratory DCs have been described to be superior at presenting antigens to T cells and initiating adaptive responses compared to LN‐resident DCs [55]. The trafficking of DCs is tightly regulated by CCR7, a chemokine receptor that is induced upon activation and acquisition of antigen, and CCR7‐deficient mice exhibit impaired DC migration from LP to gLN and cannot establish oral tolerance [56, 57]. CD103^+^ and CCR7^+^ DCs are also the APCs that exert gut‐imprint of homing markers on lymphocytes to direct them back to the intestine.

In contrast, CX3CR1^+^ DCs, often derived from monocytes, play a critical role in barrier surveillance and immune modulation within intestinal LP. These cells were thought to be gut‐resident with slow turnover rates and limited migratory capacity [55]. Rather than trafficking to gLNs, CX3CR1^+^ cells sample luminal antigens and pass them to migratory CD103^+^ DCs through Connexin 43‐dependent gap junctions transfer [41, 55]. However, multiple studies have challenged the paradigm that CX3CR1^+^ DCs are strictly non‐migratory and have found that they can also migrate to the gLN under certain conditions [58]. Flow cytometry data also demonstrated that most all major DC subsets have a subpopulation that express high levels of CX3CR1 [3], suggesting that CX3CR1 does not define cell lineage but is rather a functional marker. Although traditionally considered less involved in T cell priming due to their non‐migratory nature, CX3CR1^+^ APCs have been shown to be essential for oral tolerance. While they do not initiate Treg cell induction in the gLNs, they support the expansion and maintenance of gut‐homing Treg cells in situ via IL‐10 production [52, 59, 60].

The different roles of CX3CR1^+^ DCs and CD103^+^ migDCs underscore the coupling of function and spatial organization of DC subtypes. Chemokines and adhesion molecules also shape DC positioning and retention. Integrins like αEβ7 and chemokine receptors such as CCR9 influence DC migration and retention, in addition to the aforementioned CCR7 [61]. In general, LP‐resident DCs play a more significant role in the expansion, maintenance, or final maturation of T cell fates, such as the induction of cytokine production of T cells or migration into the intraepithelial compartment, while migDCs are essential for T cell priming in the LNs. Among the migratory CD103^+^ DC subsets, CD103^+^CD11b^+^ cDC2s preferentially migrate to gLNs to promote Th2 and Th17 polarization, while CD103^+^CD11b^−^ cDC1s support Th1 and CD8^+^ T cell responses.

Another major DC type is the resident DCs (resDCs) in the gLN, which differ from migDCs in both origin and function. resDCs are seeded and maintained within lymphoid organs from early development and do not originate from peripheral sites. Phenotypically, they can be categorized into 2 subtypes: one is often CD8α^+^XCR1^+^CD11b^−^ cells, resembling migDC1s and dependent on the same lineage‐defining transcription factors and are specialized for cross‐presentation of blood‐ or lymph‐borne antigens [62]. The other is CD8α^−^CD11b^+^ cells (cDC2 lineage), which receive transfer of antigens from newly arrived migDCs [63, 64]. Functionally, resDCs play an indispensable role in maintaining homeostatic T cell surveillance and rapidly presenting systemic antigens without relying on peripheral sampling. The presence of resDCs speeds up the response time towards systemic threats. For example, a specialized group of resDCs in the lymphatic sinuses of LNs capture particulate vaccine antigens and lymph‐borne pathogens [63]. ResDCs can also directly present antigens from afferent lymph to T cells within the T cell zone of the LN [62]. Thus, along with migDCs, the complementary roles of resDCs in the gLNs ensure broad surveillance of both local and systemic antigens.

Even within the gLNs, APC populations occupy spatially distinct intranodal niches that reflect their routes of antigen acquisition and functional specialization. MigDC1s are deep in T cell zones, where they efficiently cross‐present antigens to CD8^+^ T cells, whereas migDC2s distribute more towards the periphery of the putative T cell zone and in close proximity to the B cell follicles [65]. In contrast, resDC1s concentrate near the center of T cell zones, while resDC2s are positioned outside of the T cell zones and in lymphatic/medullary regions [65, 66]. RORγt^+^ APCs are preferentially located within the paracortex [32]. Within the small intestine tissue, CD103^+^ DCs are located both in the LP and in the intraepithelial space of apical villi, whereas CD103^−^CD11b^+^ DCs accumulate mainly in the LP [37], though they have also been shown to occupy the intraepithelial space [67], and higher resolution analysis is needed for mapping the locations of this DC subset in the intestine. Overall, this spatial organization underscores thecomplementary roles of DCs in integrating peripheral and systemic antigen surveillance.

Finally, a unique lymphoid tissue in the gut is the PP. It is notably one of the most critical sites for inducing and regulating intestinal immunoglobulin (Ig)A responses. PP DCs, particularly cDC2s, and the T cells they activate play an indispensable role in this process [2, 68]. PP DCs further specialize in antigen sampling via M cells that transcytose luminal antigens into the subepithelial dome where DCs reside [2, 69]. These DCs can subsequently engage B cells in germinal centers, promoting in situ class switching to IgA and contributing to the establishment of mucosal humoral immunity.

In sum, DC specialization by distribution in space is a powerful way to ensure broad sensing and response to the fluctuating and complex gut environment. More research will be needed to understand what determines the ratios of APC subtypes in each branch of the digestive system. Another dimension that warrants further research is the spatial positioning of APC subtypes within the tissues, especially given the identification of newer functional subsets such as cDC2A versus cDC2B and RORγt^+^ APCs. As we will discuss shortly, this could crucially influence adaptive immune outcomes.

### 2.4 Division of Labor for Different Antigen Types

DCs in the intestinal environment face a diverse array of antigens ranging from breakdown products, intact dietary macromolecules and soluble proteins to whole bacteria and dying host cells. The ability of DCs to distinguish and process these inputs is tightly linked to the mode of antigen uptake (Figure 2) and the presence of contextual signals such as PAMPs (pathogen‐associated molecular patterns) and DAMPs (damage‐associated molecular patterns). Soluble antigens, including dietary proteins or self‐proteins secreted by the gut epithelium, are sampled under non‐inflammatory conditions and taken up by LP DCs through non‐selective macropinocytosis or, in some cases, receptor‐mediated endocytosis [70]. Depending on the size and polarity of the antigens, they can also make it through the intestinal epithelium tight junctions [71] and be sampled by LN resident APCs via lymphatic delivery. In the steady state, the default immune response in the gut favors tolerance, that is pTreg cell induction, a process dependent on TGF‐β and RA, though an anergic state (FR4+CD73+ cells) is also induced [50]. This process has historically been attributed to CD103^+^ DCs; while this remains true for self‐antigens [50], recent studies show that RORγt^+^ APCs play a critical role in pTreg cell responses to dietary antigens. However, the mechanism by which RORγt^+^ APCs acquire antigens remains unclear. One possible explanation is that, like resDCs, RORγt^+^ APCs primarily take up soluble lymph‐borne antigens or receive antigen transfer from migDCs. Notably, dietary antigens are likely digested into small peptides in the stomach and small intestine and are likely more easily accessible to LN resident APCs; for example, via acellular lymphatic delivery, rather than intact proteins such as self‐antigens. Overall, it appears that soluble antigens can be loaded onto multiple APC subsets, meaning that there is no strict “single type of antigen to single type of APC” rule of division of labor for soluble antigens. However, there do seem to be constraints: dietary antigens, or at least OVA, are not loaded onto migratory cDC2Bs [33], while soluble gut‐epithelium derived antigens are not loaded onto RORγt^+^ APCs in a functionally relevant amount [50]. How broad or restricted the repertoire of APCs is that a soluble antigen is loaded onto when it is derived from a commensal or a pathogen has not been as comprehensively studied. It will also be interesting to understand whether all dietary antigens behave like OVA, which is the primary model dietary antigen used in tolerance studies.

In contrast, particulate or cell‐associated antigens, including apoptotic cells and whole bacteria, need to be taken up by phagocytosis via receptors such as DEC205, CLEC9A, and FcγRs [72, 73, 74]. APCs with the highest expression profile of these receptors are cDC1s, and our studies showed that presentation of dead‐cell‐associated cytosolic self‐antigens strictly depends on cDC1s [50]. Consequently, the fate of T cells that recognize cytosolic self‐antigens can only be altered by type 1, but not type 2 infections, underscoring the concept of DC division of labor, although notably, soluble antigens can be loaded onto cDC1s also [50]. The intestinal epithelium is thought to be replenished every 4 days, which means apoptotic cells and their associated antigens are present at high abundance. How much this cell turnover contributes to the selection of DC subtypes by which an antigen will eventually be presented, both through acting as adjuvant and as carrier of foreign and self‐antigen, is unknown and is generally ignored.

Finally, goblet‐cell associated antigen passages (GAPs) in the epithelium and M cells in PPs facilitate selective transport of luminal antigens to the underlying immune cells [2, 54]. GAPs, in particular, deliver soluble antigens directly to tolerogenic CD103^+^ DCs without triggering inflammatory responses, thereby supporting mucosal homeostasis. In contrast, M cell‐transcytosed materials in PPs are often more immunogenic [69], reflecting a structural basis for different immune outcomes based on antigen uptake route. However, these studies predate our knowledge of more resolved APC subsets, so how these antigen entry routes translate into APC subset loading and ultimately immune outcome has not been investigated. How anatomical positioning, cargo uptake machinery expression, and relative frequencies of APCs converge on determining which APC subsets can even access a given antigen will be an important future avenue to investigate. It will be even more complex in the microbiome space, where this matrix might look different for every class or even strain of microbe.

Overall, the emerging notion is that while there is division of labor between DC subtypes in terms of their sensing and output, this does not strictly reflect the antigen types they can take up. For antigen uptake, it is more likely that there are antigens with different breadths of APC subsets they can be loaded onto. In the case where an antigen can only be taken up by few or just one APC type, the adaptive immune outcome may be more predictable (e.g., cytosolic antigen). In cases where an antigen is accessible to a broader spectrum of APC subsets (e.g., soluble antigens), T cell fate may be dictated by the DC subsets that are most activated and/or mobilized at the same time. This can, in the case of soluble antigens, lead to a much broader spectrum of possible T cell fates that can be adopted in response to them. Whether there is a benefit to such flexibility in T cell response or it is an evolutionary tradeoff of robust tolerance induction can only be speculated on at the moment. On the other hand, this paradigm also means that if an antigen is only ever seen in the context of PAMPs or DAMPs, such as antigens derived from pathogens, even if theoretically that antigen is loaded onto multiple APC types, the immune response will be predictable: only the APCs that at the same time have the appropriate PPRs will be activated and/or mobilized. This will restrict the T cell responses, though again, this framework also explains why some microbes can elicit multiple T cell fates at the same time (e.g., Th and Tfh cells).

## 3 Alea Iacta Est.? DC Plasticity and Environmental Adaptation

While intestinal DCs are classically categorized into aforementioned discrete subsets, increasing evidence reveals their significant plasticity in phenotype, function and lineage. Environmental signals–including cytokines, microbial metabolites and metabolic cues–can dynamically reshape DC identity, at times even blurring the boundaries between subsets [20, 75, 76]. Under disease contexts like infections or colitis, DC phenotype and ratios within the gut tissue and related lymphoid tissues change drastically. Thus, intestinal DCs are highly adaptable, with plasticity central to both immune defense and tolerance. We will discuss this aspect in the following sections.

### 3.1 Plasticity in Division of Labor

Pro‐inflammatory cytokines, microbial products, and tissue‐derived mediators are capable of reshaping the phenotypic and functional identity of DCs. For example, prostaglandin E₂ and IFN‐γ synergize to reprogram human moDCs into cells that simultaneously possess enhanced migratory capacity (via CCR7 upregulation) [77] and inflammatory cytokine secretion (IL‐12, IL‐23) [78], illustrating the coexistence of previously distinct functional attributes in a single DC phenotype.

Single‐cell RNA‐seq and lineage‐tracing studies have uncovered DC populations in transitional states [9, 79, 80], suggesting that phenotypic boundaries between classical subsets may be more fluid than previously thought. For instance, intestinal CD103^+^CD11b^+^ DCs have been shown to arise from both monocyte and pre‐DC lineages [37], with distinct migratory and functional capacities depending on their origin and inflammatory context. Moreover, cDC2s—typically associated with Th17 responses—can acquire cross‐presentation capacity and promote CD8^+^ T cell priming when exposed to type I interferons or TLR3 ligands, a function traditionally ascribed to cDC1s [20].

Metabolic inputs also regulate DC plasticity. During inflammation, hypoxia‐inducible factor 1α (HIF‐1α) promotes a metabolic switch to glycolysis in DCs, enhancing their pro‐inflammatory capacity [81], while oxidative phosphorylation and fatty acid oxidation are associated with a tolerogenic state [82]. AMPK activation, for example, drives moDCs towards a regulatory phenotype marked by increased RALDH activity and Treg cell‐inducing potential [82]. These findings underscore the coupling of metabolic state to immune function in shaping DC behavior.

Plasticity is not only a feature of healthy immune adaptation but also plays a role in disease. In the inflamed gut, such as in patients with IBD, Ly6C^hi^ monocytes upregulate TLR2 and NOD and can differentiate into DC‐like populations with potent antigen‐presentation and cytokine production (IL‐6 and IL‐23) capability [83]. Upon infections with LPS‐expressing microbes, blood monocytes are recruited to the gut or gLN by the microbial signals and fully differentiate into DC‐SIGN/CD209^+^ DCs [84]. This capacity for environmental reprogramming enables the intestinal DC network to sustain immune surveillance under stress, though it also presents risks—particularly in chronic inflammation or cancer, where DC plasticity may be subverted to promote immune suppression or tolerance.

Together, these findings reveal that DCs in the intestine are not static entities but are dynamically tuned by their local environment. This functional malleability is central to their capacity to balance tolerance and immunity and presents opportunities for therapeutic manipulation through cytokine, microbial, or metabolic interventions.

### 3.2 Environmental Imprinting of Dendritic Cells

DCs in the digestive system are unique in that they are constantly exposed to environmental fluctuations, including microbes and microbial products, food antigens, and dietary nutrients. While the field has historically focused on the impact of microbial sensing in DC maturation and activation, more recent work has highlighted other tissue‐specific factors that may also influence DC behavior. This section will discuss environmental factors that influence DC behavior and function, highlighting key gaps in knowledge that remain in this active area of research.

#### 3.2.1 Tissue Imprinting of Dendritic Cells in the Digestive System

The intestinal tract is highly specialized for food digestion, with each segment having a unique role in nutrient handling and metabolism. The pancreas supports digestion through the secretion of digestive enzymes into the duodenum for the breakdown of carbohydrates, proteins, and lipids, while the liver supports fat digestion and absorption through bile acid secretion. The small intestine is responsible for nutrient absorption, while the large intestine facilitates the absorption of water and fermentation products. The tissue environment differs significantly between the small and large intestine due to differences in nutrient availability, pH and oxygen levels, and microbial burden and diversity. Notably, the abundance of cDC populations also differs between small and large intestine, with the small intestine being enriched for CD103^+^CD11b^+^ cDC2s and the large intestine for CD103^+^CD11b^−^ cDC1s [85]. In fact, each tissue in the body appears to harbor a unique relative abundance of cDC populations in both mice and humans [86, 87], although the factors dictating this remain unclear.

**FIGURE 3:**
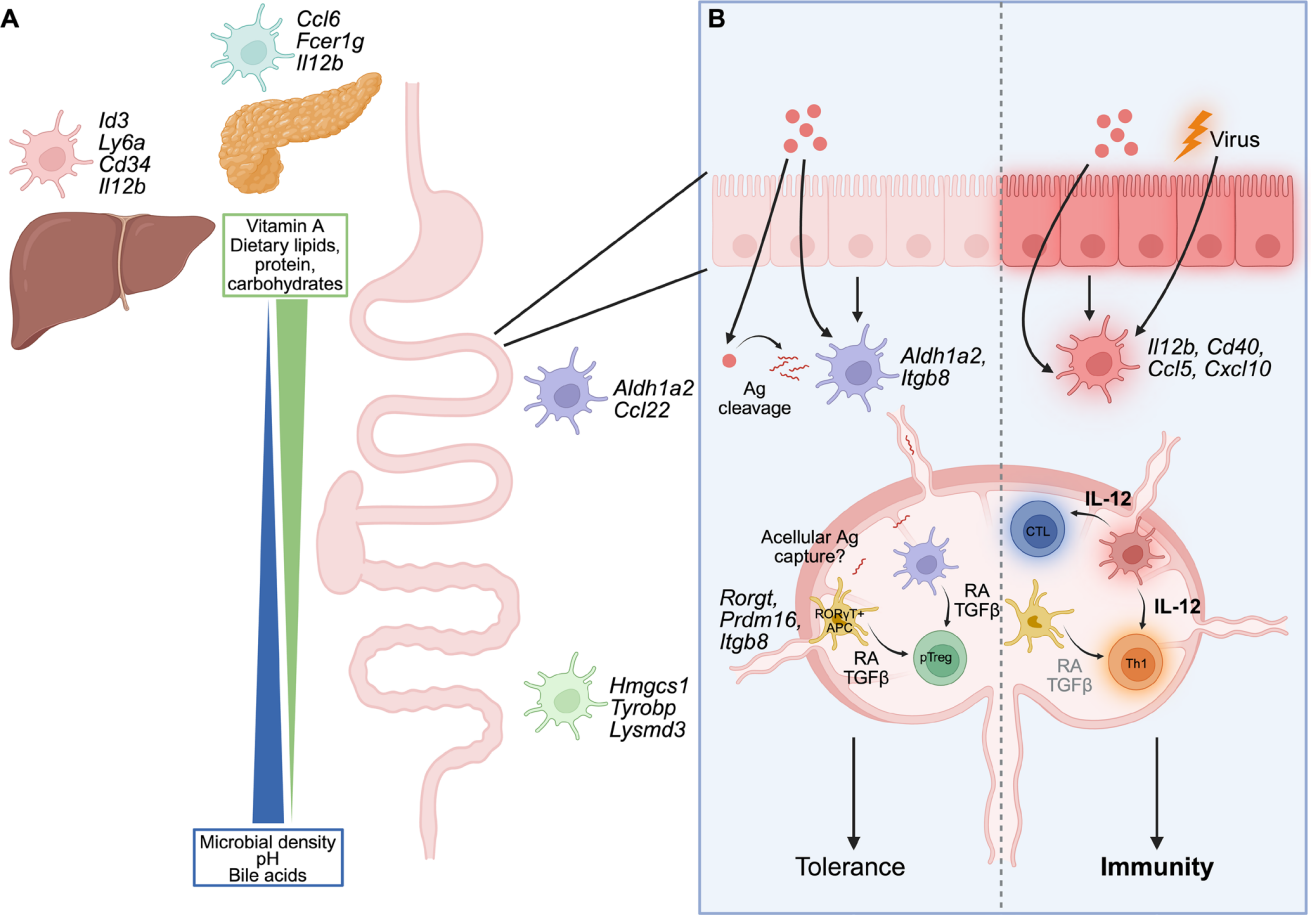
cDC1s display tissue‐specific signatures and adapt to tissue environments. (A) Summary of cDC1s transcriptional signatures in the digestive organs. cDC1s differ along the gut, with *Ald1a2* and *Ccl2* being enriched in duodenal cDC1s and playing a role in pTreg cell differentiation. Colonic cDC1s are enriched for transcripts such as *Hmgcs1*, which is involved in cholesterol biosynthesis, and *Lysmd3*, which encodes a pattern recognition receptor for chitin. When compared to duodenal cDC1s, liver and pancreatic cDC1s are enriched for pro‐inflammatory genes including *Il12b* which plays a role in Th1 differentiation. *Fcer1g* is specifically enriched in pancreatic cDC1s, while liver cDC1s are enriched for transcripts reflecting an immature state including *Cd34* and *Ly6a*. (B) Intestinal cDC1s adapt to inflammatory environments and differentially instruct T cell differentiation. At homeostasis, cDC1s integrate signals derived from the tissue environment (nutrient levels, epithelial cell‐derived factors, etc.) while taking up soluble antigens. High expression of *Aldh1a2* and *Itbg8* facilitate tolerogenic immune responses to soluble antigens in the lymph node through RA and TGFβ production. RORγt^+^ APCs also play a role in this response when the antigen source is dietary or commensal, and cDC1s and RORγt^+^ APCs may even cooperate for maximal pTreg cell induction. Upon intestinal viral infection, cDC1s adopt a pro‐inflammatory signature characterized by expression of *Il12b*, *Cxcl10*, *Cd40*, and *Ccl5* which all contribute to Th1 differentiation, CD8^+^ T cell activation and loss of oral tolerance.

The work of our lab and others has shown that the duodenum uniquely harbors a significant population of CD103^+^CD11b^+^ migratory cDC2s which are nearly absent in the liver and pancreas [88] and very lowly abundant in the kidney and lung [89]. This population of cDC2s is dependent on RA and plays a key role in driving Th17 cell differentiation [90], perhaps explaining their distinctively high abundance in the small intestine. In addition to differences in DC subset frequency, DCs adopt tissue and region‐specific gene expression profiles. RNA sequencing (RNA*seq*) analysis of the total DC population isolated from duodenum, jejunum, ileum, cecum, and colon has revealed segment specific transcriptional signatures, highlighting that the intestinal segments differ not only physiologically but also immunologically [91]. While there have not been studies that comprehensively compare the transcriptome of a single DC subset across tissues, we have found that CD103^+^CD11b^−^ migratory cDC1s from the duodenum, liver, and pancreas are transcriptionally unique [88]. Notably, cDC1s from the liver and pancreas are enriched for genes related to lymphocyte activation and pro‐inflammatory processes such as *Il12b*, *Il15ra*, and *Ccl5* when compared to the duodenum, which in contrast is enriched for *Aldh1a2*. Interestingly, liver cDC1s also express genes reflective of an immature state such as *Id3* and *Cd34* (Figure 3A). However, comparative analysis of additional tissues is needed to fully understand whether the pro‐inflammatory signatures shared by liver and pancreatic cDC1s are similar to other tissues, thus suggesting that intestinal cDC1s are uniquely tolerogenic. Few studies in the field have taken a global cross‐tissue approach to understand the tissue‐specific imprinting of dendritic cells across the body. Work in our lab suggests that the small intestine may be a particularly unique environment, likely due to the crucial role it plays in food tolerance and host metabolism. Comprehensive analysis of DCs across the body would provide insight into the role the tissue environment plays in DC function. Still, collectively these results indicate that adaptive immune responses may be influenced both by the relative abundance of cDC subsets in tissues as well as tissue specific imprints on cDCs.

While migratory cDCs differ based on the tissue in which they initially sit, they also harbor distinctions based on the LNs to which they migrate. The difference in cDC landscape between the small and large intestine is largely retained in the gLNs, where each segment drains to an anatomically distinct LN or set of LNs [85]. All migratory cDCs undergo significant changes as they migrate to the LNs, including upregulation of CCR7 to facilitate entry into lymphatic vessels and, upon inflammatory stimuli, upregulation of costimulatory molecules and MHC class II. However, migratory cDC1s and cDC2s are transcriptionally distinct in each gLN [39], demonstrating that cDCs in each gLN are predisposed to induce different responses that reflect the immunological needs of the tissue they drain. In line with this idea, the fate of oral antigen specific T cells differs in each gLN leading to a peripheral Treg cell gradient that is highest in the duodenum draining LNs and decreases in the distal intestine draining LNs [39]. One factor contributing to this is the relatively high expression of *Aldh1a2* in upper small intestine DCs. RA produced by DCs promotes pTreg cell differentiation while simultaneously inhibiting Th17 differentiation [92], making it an important molecule for oral tolerance.

Similarly, we have studied the impact of tissue‐specific cDC imprints on adaptive immune outcomes in the context of lymph node sharing between the duodenum, liver, and pancreas [88]. We have found that cDC1s migrating from the duodenum, liver, and pancreas largely retain the gene expression signatures that were imprinted by tissue of origin, and this has consequences for the priming of pancreatic antigen‐specific T cells based on the relative contribution of drainage from the duodenum versus liver. This suggests that differences in adaptive immune outcomes in LNs may be at least in part due to the retention of tissue‐specific signatures that were imprinted prior to migration, in combination with differences in stromal cells in each LN [39, 93]. However, it is still unknown what tissue‐derived factors cDCs are responding to that influence their gene expression profiles and ultimately their interactions with T cells.

While much of the aforementioned work has focused on migratory cDC populations, little has been done to understand the LN specific specialization of resident cDC populations. This may be particularly important for the LNs draining the digestive organs as the gut and liver are the body's major lymph output organs [94]. Lymph composition varies significantly by tissue, which could differentially influence resident cDCs that are constantly exposed to afferent lymph [95]. Notably, gLN‐resident DCs are capable of inducing pTreg cells, and their tolerogenic potential is imprinted and maintained by LN stromal cell populations [93]. Our work suggests that LN environments may play a significant role in T cell fate both at homeostasis and upon perturbation. Future work could define the role that resident DCs play in setting the immunological tone of each LN and the consequences of this on tissue specific immune responses.

#### 3.2.2 Dendritic Cell Reprogramming and Distribution Upon Microbial and Tissue Perturbations

While intestinal cDC1s have high tolerogenic potential at steady state through secretion of RA and TGFβ, this subset can be reprogrammed to promote immunity upon exposure to intracellular microbes or tumors. Early studies demonstrated the ability of cDC1s to produce IL‐12 in response to bacterial or viral PAMPs [96, 97, 98, 99], which can be further boosted by CD40 ligation by T cells [100] (Figure 3B). While type I interferons and other signals can poise cDC1s to respond more rapidly, IL‐12 production and subsequent Th1/CTL differentiation require PAMP recognition by PRRs [101]. The requirement for cDC1s in adaptive immune responses to viruses and tumors has been demonstrated with genetic models of cDC1 depletion [102, 103, 104]. In addition to cytokine production, cDC1s produce chemokines such as CXCL10 that facilitate their interactions with T cells during priming in the LN [105].

More recent work has provided a more nuanced picture of the transcriptional state that intestinal cDC1s adopt upon direct infection with microbes or interactions with infected or transformed cells. RNA*seq* of migratory cDC1s in gLNs upon infection with the intestinal reovirus T1L showed a significant change in gene expression. Among the upregulated genes were interferon stimulated genes, antigen processing and presentation genes, and genes involved in the priming of CD4 and CD8 T cells such as *Il27*, *Cd40*, and *Cd274* [106] demonstrating a proinflammatory switch of cDC1s (Figure 3B). Interestingly, T1L does not directly infect cDC1s but rather infects intestinal epithelial cells that ultimately die and slough into the intestinal lumen [107].

Intestinal cDC1s also receive signals from the gut microbiota that shape their function and transcriptional state. The previously mentioned study also demonstrated the transcriptional adaptation of cDC1s to the gut microbiota, specifically the commensal protist *T. arnold*. cDC1s from *T. arnold* colonized mice had decreased expression of *Cdk19* and *Clec7a*, which have been shown to negatively regulate tolerogenic cDC responses. This change in cDC1 gene expression profile correlated with an increase in dietary antigen specific Treg cells in the gLNs even in the presence of T1L [106]. These results suggest that cDC1s can not only respond to viruses for which they are specialized but also integrate signals from other commensal organisms that may impact adaptive immune outcomes. Another study found that cDC1 deficient mice develop an outgrowth of commensal *Cyryptosporidium*, which normally induces a Th1 response that controls its abundance in the gut [108].

Our studies on lymph node sharing in the upper digestive system have demonstrated that virally induced “switched” cDC1s can not only influence the behavior of T cells but possibly also surrounding cDC1s. To investigate this, we adoptively transferred pancreatic beta cell antigen‐specific CD4 (BDC2.5) and CD8 (NY8.3) T cells to non‐obese diabetic mice and used the intestinal reovirus T1L to probe the impact of duodenal infection on pancreatic immune responses. At homeostasis, duodenal cDC1s likely have a tolerogenic influence on pancreatic cDC1s, as shown by the increase in FOXP3^+^ BDC2.5 T cells in pancreatic LNs shared with the duodenum compared to those shared with the liver [88]. However, upon intestinal viral infection, we observed a decrease in FOXP3^+^ BDC2.5 cells and an increase in TBET^+^ BDC2.5 T cells and Granzyme B^+^ NY8.3 T cells. We hypothesized that intestinal cDC1s may either (1) transmit information to cDC1s carrying pancreatic antigen through soluble factors such as IL‐12 or type I interferons or (2) transmit information to BDC2.5 and NY8.3 T cells directly through proximity. Interestingly, while we were unable to show directly that pancreatic cDC1s are impacted by intestinal cDC1s due to difficulty in pancreatic cDC1 tracing methods, we found an increase in IL‐12p40^+^ liver‐derived cDC1s upon intestinal viral infections, suggesting potential communication between inflammatory cDC1s in the LN. Thus, paracrine signaling between cDC1s upon infection may contribute to immunity to viral infection, though it is possible that pancreatic and hepatic cDC1s receive imprinting in the LNs through other sources like lymph or stromal cells that were altered first. It is unknown whether cDC2s have a similar mechanism of paracrine communication upon infection or tissue stress.

Apart from pathogens, cDC1s play context dependent roles in settings of tissue damage or stress such as cancer. Given the role of cDC1s in cross‐presentation and CD8 T cell priming, they are generally thought to promote anti‐tumor immunity. However, the function of cDC1s in the context of colon cancer is still largely unknown. Single cell RNA*seq* (scRNA*seq*) of cDC1s in human CRC tumors as well as murine MC38 syngeneic tumors revealed that they are present primarily in an immature state with relatively low expression of *Ccr7*, *Il12b*, and *Cd40* [109]. αCD40 treatment led to an increase in cDC1 frequency and activation, resulting in increased tumor clearance. More extensive profiling of cDC1s in normal colonic tissue compared to colorectal tumors would elucidate the impact of the tumor microenvironment on cDC1 function. Interestingly, a unique subset of DC has been identified in several cancer types including colorectal cancer and hepatocellular carcinoma that is characterized by high expression of LAMP3 and CCR7 [109, 110]. These DCs are transcriptionally distinct from both cDC1s and cDC2s and appear to be mature DCs with high capacity for LN migration. More work has been done to determine the role of cDC1s in pancreatic ductal adenocarcinoma (PDAC) tumors. When compared to lung adenocarcinoma, cDC1s in PDAC are present at a reduced number and have a decreased capacity to take up tumor antigens [111]. This dysfunctional state is acquired over time as tumors develop [112], suggesting that factors in the PDAC microenvironment may condition cDC1s towards a functionally immature state or exclude them from tumors. Overall, cDC1s may adopt distinct functional states depending on the tumor environment.

Similar to cDC1s, cDC2s can adopt a unique transcriptional profile upon infection or tissue perturbation. cDC2s play a crucial role in the differentiation of Th2, Th17, and Tfh cells, each requiring a unique set of signals to drive polarization. This has prompted extensive work into the requirements and driving forces that dictate which T helper subset cDC2s will instruct based on the signals they receive from the environment.

The role of gut cDC2s in Th2 differentiation has been most well characterized upon intestinal helminth infection, which elicits a strong Th2 response that requires cDC2s [13]. While the precise signals that cDC2s provide to CD4 T cells to instruct Th2 differentiation are incompletely understood, cDC2s can be programmed towards eliciting a Th2 response by a variety of signals such as alarmins [113] and neuropeptides [114], and other immune cells may provide supportive signals to direct naïve T cells towards a Th2 fate [115]. A recent study investigated the response of cDC2s to helminth infection by performing scRNA*seq* of migratory cDCs upon infection with *S. venezuelensis* or *H. polygyrus*, both helminths with strong tropism for the duodenum [33]. They identified 3 separate clusters of cDC2s, one of which was a “Th2 inducing” subset as it significantly expanded upon helminth infection. This subset compared to the other cDC2s was enriched for genes that were previously identified to be important for Th2 differentiation, including *Ccl24*, *Cd1d1*, and *Stat5a*, and overall was enriched for gene signatures involved in T cell priming and DC maturation. A similar profile was identified in cDC2s in the duodenum lamina propria upon helminth infection, suggesting cDC2s adopt this state in the tissue prior to migration to the gLNs. While many studies have focused on migratory cDC2s in Th2 differentiation, LN resDCs may also play an important role [116]. Interestingly, studies with *S. mansoni* eggs showed that while CD103+ CD11b+ cDC2s are necessary for Th2 differentiation in the small intestine (consistent with helminth studies), CD103− CD11b+ cDC2s are the most significant population priming Th2 cells in the colon [117]. While the factors underlying this difference are unknown, this underscores the importance of comparing cDCs from multiple tissues as they may have distinct functions.

Studies with intestinal helminths have demonstrated not only how changes to the gene expression of cDC2s can impact immune responses but also how a change in the composition of the DC compartment can contribute. Infection with the helminth *S. venezuelensis* leads to a significant increase in the abundance of both CD103^+^ and CD103^−^ cDC2s in the duodenal LN, resulting in a drastic decrease in CD103^+^ cDC1s [39]. Given the role of cDC1s in pTreg cell induction, either through direct instruction of naïve T cells or facilitating the capture of antigen by LN‐resident RORγt^+^ APCs, this exclusion of migratory cDC1s from the duodenal LN may explain the partial loss of oral tolerance upon helminth infection. Importantly, the mechanism by which oral tolerance is broken is highly dependent on the type of infection; upon viral infection, reprogramming of tolerogenic cDC1s is responsible rather than a lack of cDC1 migration or LN entry. However, the idea that DC compartment composition can impact immune outcome may be relevant beyond the context of intestinal infection.

cDC2s also instruct the differentiation of Th17 cells, which are particularly important in the intestine to maintain tolerance to commensal microbes and provide immunity to intestinal bacterial and fungal pathogens. Although the full transcriptional program adopted by cDC2s upon microbial exposure is still not entirely clear due to the lack of RNA*seq* data available, many studies have shown that cDC2s secrete IL‐1β, IL‐6, IL‐23, and TGFβ in response to TLR engagement to instruct Th17 cell differentiation.

Like cDC1s, cDC2s also sense and respond to the intestinal microbiota, but they express a different repertoire of PPRs from cDC1s. cDC2s are relatively enriched for several PRRs that may facilitate their interactions with commensal bacteria including TLR4, TLR6, and NOD2 [3]. cDC2s in intestinal PPs also express the C‐type lectin receptor *Mincle*, a PRR that recognizes a variety of ligands, some of which are expressed by microbes including *Lactobacillus* strains [118]. *Mincle* expression by cDC2s was required for IL‐6 and IL‐23 secretion and subsequent Th17 cell differentiation in response to the microbiota, suggesting that this axis may be important for mucosal barrier integrity [119]. Similarly, migratory cDC2s load and present antigen derived from commensal segmented filamentous bacteria (SFB) to prime Th17 cell responses in an IL‐6‐dependent manner [120, 121]. Notably, the bacterial strains described in these studies are known to associate closely with the intestinal epithelium [119, 122], suggesting that microbes that have a high likelihood of breaching the intestinal barrier are more readily (or exclusively) sensed by DCs and other innate immune cells. Future studies may shed light on what microbial features dictate their interactions with DCs in the gut.

Recent work has added further nuance to the “reprogramming” of cDC2s in that it was discovered that ontogenically distinct cDC2 subsets, cDC2A and cDC2B, drive Th2 vs. Th17 cell responses: Notch2‐dependent cDC2s (now termed cDC2A) are essential for host defense against *C. rodentium* , an attaching and effacing bacterial pathogen that elicits a strong Th17 cell response in the colon, due to their production of IL‐23 that stimulates IL‐22 production by ILC3s [18]. Conversely, KLF4‐dependent cDC2Bs were found to be required for Th2 cell responses to *S. mansoni* as conditional deletion of *Klf4* in CD11c expressing cells resulted in decreased survival [15]. DC‐specific deletion of *Klf4* did not impair responses to *C. rodentium* , again suggesting that a different subset of cDC2s is required for type 3 immune responses. Future work could identify which subset(s) is responsible for Tfh cell generation.

While cDC2s are important for mucosal homeostasis, they may also be involved in intestinal diseases including inflammatory bowel disease (IBD). While cDC1s are thought to play beneficial roles at least in mouse models of IBD [123], cDC2s are implicated in aberrant immune responses to the microbiota that are characteristic of IBD immunopathology. Notably, current therapies for IBD include antibodies that target IL‐23 either by blocking the activity of IL‐12p40, a subunit of both IL‐12 and IL‐23, or blocking solely IL‐23 with antibodies targeting the IL‐23p19 subunit [124]. However, more work needs to be done to delineate the role of cDC2s and CD11b+ macrophages, which heavily infiltrate the inflamed mucosa in IBD patients and can also contribute to inflammatory cytokine production [125]. cDC2s may also play a role in food allergy due to their ability to promote Th2 polarization and subsequent class switching of B cells to IgE, although their specific role in promoting food allergy is still an active area of investigation [126]. The recently proposed paradigm in which two subsets of cDC2s (cDC2a and cDC2b) direct Th2 versus Th17 cell responses may have implications for these intestinal diseases. The equivalent cells of these two subsets have been described in humans [16], suggesting a similar division of labor between cDC2 subsets is conserved across species. Based on the phenotype of food allergy compared to IBD, it appears that food allergy may be driven by cDC2B cells while IBD is driven by cDC2A cells. It is possible that different flavors of antigen are loaded onto different cDC2 subsets depending on the immunological context of the intestine. Future work could delineate the role of each cDC2 subset in different diseases and determine how microbial, dietary, and self‐antigens are handled by different DCs.

Many studies have shown the increase in percentage of cDCs that produce proinflammatory cytokines upon microbial infection. However, it is still unclear for both cDC1s and cDC2s if these cells are truly reprogrammed into proinflammatory cells upon infection or if newly influxing DCs mature with a different profile upon entry into a perturbed tissue. This would require profiling of pulse‐chased DCs upon infection.

#### 3.2.3 Metabolic Adaptations of Intestinal Dendritic Cells

The metabolic programming of DCs is increasingly recognized as another key determinant of their immune functions. The intestine is a unique environment for DCs due to the abundance of dietary nutrients and commensal bacteria, both of which contribute to the metabolic environment of the tissue. DCs, like all cells in the gut, experience the highest exposure to dietary molecules in the entire body. A key nutrient driving intestinal DC differentiation and function is Vitamin A and its bioactive derivative RA [127]. DCs in the small intestine and its draining LNs experience particularly high exposure to Vitamin A, as it is a lipid soluble vitamin that is transported in large parts by small intestinal chylomicrons. Indeed, CD103^+^ cDC1s in the small intestine and draining LNs express higher levels of *Aldh1a2* than those in the colon [39], suggesting a ligand‐dependent feed forward loop of RALDH levels. However, in the colon, short chain fatty acids derived from the diet and intestinal microbiota also increase the expression of *Aldh1a2* by DCs and thus colonic DCs still exhibit higher production RA than DCs in most non‐intestinal locations, such as spleen [128]. RA is essential for imprinting gut‐homing markers on T cells, promoting the differentiation of Treg cells [3] and class switching of B cells to IgA^+^ B cells [129, 130]. The high availability of both Vitamin A and food antigens in the upper small intestine underscores the evolutionary benefit of this transcriptional adaptation of DCs to maintain tolerance to dietary antigens. Interestingly, RA can promote a pro‐inflammatory Th1 response to dietary antigen in the context of high intestinal IL‐15 expression [131], demonstrating that DCs integrate multiple cues that ultimately decide their impact on T cell fate. Thus, the same antigen can elicit tolerance or immunity depending on the environmental cues that are received by and the activation status of the presenting DC.

Another key enzyme, indoleamine 2,3‐dioxygenase (IDO), metabolizes dietary tryptophan and promotes Treg cell induction while suppressing effector T cell responses, often in response to IFN‐γ or microbial signals [132, 133]. DCs, in particular cDC1s, are enriched in *Ido1* [132, 133]. cDC1s that lack *Ido1* have increased expression of *Tnfa* and decreased expression of *Pdl1*, resulting in increased T cell proliferation and Th1 polarization. Interestingly, LPS stimulation increases expression and activity of *Ido1* by cDC1s, leading to the release of tryptophan metabolite L‐kynurenine. L‐kynurenine acts on cDC2s via aryl hydrocarbon receptor to acquire IDO1 expression and regulate IL‐6 secretion, demonstrating the ability of IDO1 to facilitate crosstalk between DC subsets [134]. Furthermore, microbes also utilize dietary tryptophan and generate bioactive catabolites such as aryl hydrocarbon receptor (AHR) ligands [135], which in turn can act on DCs [136]. Thus, both Vitamin A and tryptophan metabolism in the intestine are examples of how DC function is tuned by the interplay between nutrient availability and microbiome.

In addition, microbial lipid metabolites, specifically oxidized fatty acids, have been shown to facilitate pTreg cell differentiation [137]. However, direct treatment of DCs with microbial lipids did not alter RALDH activity, suggesting that these lipids promote pTreg cells through a mechanism that is distinct from that of SCFAs [137].

Dietary macromolecules have also been shown to influence intestinal DCs and their ability to promote pTreg cell differentiation to dietary antigens. In the crudest approach, a 36 h fast, it was shown that such nutrient deprivation leads to a severe loss of migratory DCs in the lamina propria and LNs, reduced RALDH activity and pTreg cell induction, and the effect of this fast persisted 48 h after refeeding [138]. Glucose and arginine were the main nutrients that were needed to overcome this deficit, likely because of their potency in restoring mTOR signaling in DCs [138]. Several studies have utilized the antigen‐free diet, which lacks macromolecules such as proteins and starches. Mice placed on this diet have decreased numbers of CD103+ cDC2s in the small intestinal LP, and these cells have lower expression of *Aldh1a2*, *Ido1*, and *Tgfb1* than in mice on a complex diet. This correlated with a decreased number of pTreg cells in the siLP compared to SPF and GF mice on a normal chow diet [128, 139]. Interestingly, only LP but not gLN DC RALDH activity was affected by the antigen‐free diet, and the key regulatory component was glucose. However, it is largely unknown how dietary macromolecules alter the overall transcriptional profile of intestinal DCs. RNA*seq* of DCs from the upper SI of mice fed a protein‐free diet revealed a downregulation of genes involved in T cell interactions and priming (*Cd86*, *Ccr7*, and *Cd83*) compared to DCs from control mice, but this effect was likely largely due to the impact of dietary proteins on the gut microbiome rather than direct impact on DCs [140]. Because the diets used in these studies also impact microbial composition [139, 140], it may be difficult to disentangle the precise role of dietary nutrients on DCs and that of the microbiota. It is also unclear if RORγt^+^ APCs are impacted by dietary nutrient availability. Thus, both dietary and microbial metabolites can tune the functions of intestinal DCs which ultimately has consequences for T cell homeostasis.

Bile acids are another component of the digestive system that can impact DC function. A small portion of bile acids secreted into the small intestine are metabolized by the microbiota into secondary bile acids. One study identified two secondary bile acids, ω‐muricholic acid (ω‐MCA) and isolithocholic acid (isoLCA), that enhanced FOXP3 expression in naive CD4 T cells when cultured in vitro with DCs [141]. RNA*seq* analysis of DCs treated with isoLCA revealed an upregulation of genes involved in antigen processing and presentation including *Ciita* and *H2ab* and downregulation of genes involved in the detection and response to inflammatory cues include several TLR genes and ISGs. One study showed that secondary bile acids entering circulation can suppress NFkB signaling in DCs in a model of experimental uveitis [142], suggesting that these molecules may also impact liver and pancreatic DCs where the concentration of secondary bile acids may be even higher than systemic circulation. Finally, pharmacological agents, like dexamethasone, rapamycin, and supplemental Vitamin D3 have been shown to polarize DCs towards a more tolerogenic phenotype [143].

## 4 Open Questions, Future Directions and Potential of Intestinal DC Research

The past two decades unveiled the complexity of the DC landscape in the digestive system, and studies comparing DCs from different niches within the digestive tract and under different environmental conditions made us appreciate the local adaptation capacity of DCs. We also know that beyond the gut, gastrointestinal and LN DCs differ from those in the spleen [128], or conversely that intestinal and pulmonary DCs share some features like RALDH activity and impose the same homing receptor expression on T cells [144]. Our study focused on cDC1s shows that the transcriptional profile is different even in close by, developmentally linked organs like liver, duodenum and pancreas [88]. Yet, more comprehensive and systematic analyses of DC subtypes across organs would be required to fully appreciate the degree to which DCs adapt to the organ in which they reside. ScRNA*seq* approaches have been taken to profile tissue immune landscapes; however, since DCs are comparatively rare and harder to isolate, they tend to be underrepresented in such studies, and the effort would involve greatly enriching for DCs. This knowledge is important to not only better understand tissue‐specific adaptive immunity, but also to predict the effectiveness or side effects of potential therapeutic approaches based on targeting DCs. Additionally, much of what we know about gastrointestinal DCs stems from research in mice, while our knowledge in humans is much more limited. The fundamental principles of the existence of multiple DC subsets in the gut and draining LNs [44, 145] as well as site‐specific transcriptional adaptations [88] hold true in humans, and overall, there seems to be a human equivalent to all DC and APC subsets found in mice, including cDC1, cDC2, moDCs, migratory DCs and RORγt^+^ APCs. However, more dynamic biology such as how these cells respond to perturbations or what their turnover rates are in humans, is unknown and challenging to study. Again, this would be insightful in order to develop or improve therapeutics targeting DCs. One major prediction from the observation of DC plasticity in mice is that it is likely more efficient and precise to target a DC function, rather than lineage. However, the potency of even such a strategy; for example, blocking or activating a receptor, may heavily depend on where DCs anatomically reside. Thus, we are likely only on the cusp of how DCs can be harnessed to improve gastrointestinal health.

## Conflicts of Interest

The authors declare no conflicts of interest.

## Data Availability

No new data are generated in this review.
